# Enterovirus D68 Outbreak in Children, Finland, August–September 2022

**DOI:** 10.3201/eid2906.221795

**Published:** 2023-06

**Authors:** Ville Peltola, Riikka Österback, Matti Waris, Lauri Ivaska, Paula A. Tähtinen, Miia Laine, Tytti Vuorinen

**Affiliations:** Turku University Hospital and University of Turku, Turku, Finland

**Keywords:** Enterovirus, EV-D68, children, pneumonia, respiratory tract infection, wheezing, respiratory infections, viruses, Finland

## Abstract

We observed an intense enterovirus D68 outbreak in children in southwest Finland in August–September 2022. We confirmed enterovirus D68 infection in 56 children hospitalized for respiratory illnesses and in 1 child with encephalitis but were not able to test all suspected patients. Continuing surveillance for enterovirus D68 is needed.

Widespread enterovirus D68 (EV-D68) epidemics occurred in the United States and in other parts of the world in 2014, 2016, and 2018 (*1*,*2*). Low-level EV-D68 detection was reported in the United States during the COVID-19 pandemic in 2020 (*3*). EV-D68 re-emerged in autumn 2021, prominently in Europe but also to some extent in the United States, and again in 2022, when reports emerged from the United States and Europe about a substantially increased circulation of EV-D68 associated with acute respiratory illnesses in children (*4*–*6*). Previous EV-D68 epidemics in Europe and the United States were associated with severe lower respiratory tract infections, wheezing illnesses, and, more rarely, acute flaccid myelitis or other neurologic presentations in children (*7*,*8*). The clinical manifestations during the outbreaks in 2022 have not been described in detail in the literature. 

In Finland, EV-D68 cases were detected in 2014 and sporadically before and after that, but circulation of the virus has not been seen during recent years. All laboratory findings of enterovirus in Finland are reported in the Finnish National Infectious Diseases Register ,and some enteroviruses are typed. Hospitalizations of children because of respiratory illnesses increased rapidly in southwest Finland in August 2022. We screened children (≤16 years of age) admitted to Turku University Hospital, Turku, Finland, for respiratory or central nervous system illness associated with enterovirus (confirmed by reverse transcription PCR [RT-PCR]) and identified EV-D68 by type-specific RT-PCR and sequencing. We describe the outbreak and report the clinical features of 57 children hospitalized with EV-D68 in August–September 2022.

## The Study

Turku University Hospital is the only hospital providing inpatient care for children in the Southwest Finland Hospital District, covering a population of 470,000 people, including 77,000 children and adolescents ≤16 years of age. The hospital also serves as a tertiary facility for a larger area, but our study did not include patients referred from other hospitals. Hospital policy requires that all children and adolescents with symptoms of infection at admittance be screened by a rapid RT-PCR test for SARS-CoV-2, influenza A and B, and respiratory syncytial virus (RSV). A laboratory-developed triplex RT-PCR test for enterovirus, rhinovirus, and RSV (*9*) or a 16-plex RT-PCR test for respiratory viruses including enterovirus (Allplex Respiratory Panels 1–3; Seegene, https://www.seegene.com) is performed by using nasopharyngeal swab or cerebrospinal fluid specimens for patients with a respiratory tract or central nervous system illness and negative screening test (Appendix). 

**Figure 1:**
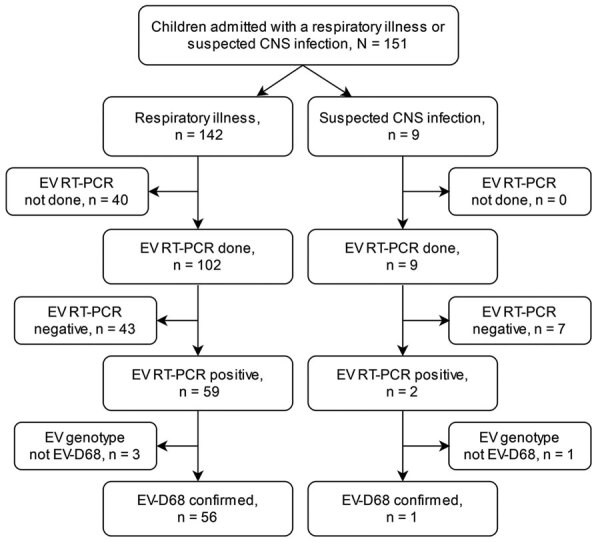
Flowchart of the enterovirus D68 (EV-D68) testing process of children hospitalized with respiratory illness or suspected central nervous system (CNS) infection at Turku University Hospital, Turku, Finland, during August 1–September 30, 2022. EV, enterovirus; RT-PCR, reverse transcription PCR.

When we noticed an enterovirus outbreak, we aimed to genotype viruses in all detected cases. First, we subjected specimens positive for enterovirus to typing based on partial enterovirus viral protein (VP) 1 gene sequencing after seminested PCR amplification (*10*). We observed a high prevalence of EV-D68, but the amplification succeeded only in specimens with high virus load (low cycle threshold value). Therefore, we applied an RT-PCR assay with EV-D68 VP1 gene-specific primers (*11*). Because a portion of the specimens remained untyped, we subjected them to a nested amplification and sequencing for the VP4/2 gene region (*9**,**12*) (Figure 1; Appendix)

**Figure 2:**
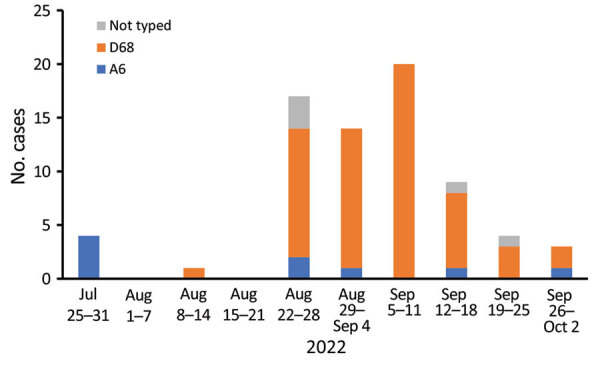
Weekly number of enterovirus D68, coxsackievirus A6, and nontyped enteroviruses in children hospitalized with enteroviral illness in southwest Finland during July 25–October 2, 2022.

In August 2022, hospitalization and intensive care unit admittances of children for wheezing illnesses and pneumonia increased rapidly at our hospital. EV-D68 was documented in most children who tested positive for enterovirus (Figure 2). During the study period of August 1–September 30, 2022, a total of 57 children were hospitalized with a confirmed EV-D68 infection. The weekly number of new EV-D68 cases ranged from 7 to 20 during the 4-week period of August 22–September 18, 2022, and decreased thereafter.

The median age of the 56 children hospitalized with EV-D68 respiratory illness was 4.23 years (interquartile range 2.42–6.15 years), and most (66.1%) were male (Table). Before their most recent illness, 26.8% of the children had confirmed asthma or had been prescribed inhaled corticosteroids for suspected asthma. The primary reason for hospitalization was wheezing illness for 78.6% and pneumonia for 16.1%. The mean length of hospital stay among the children was 2.75 days (SD 2.05 days). More than half needed supplemental oxygen or other respiratory support, and 7 (12.5%) were admitted to the intensive care unit. The study population included 1 case of encephalitis and no cases of acute flaccid myelitis. The child with encephalitis manifested ataxia as the predominant symptom and did not need intensive care; EV-D68 was detected in the nasopharyngeal specimen but not in cerebrospinal fluid. All children recovered fully, and only 1 child was re-admitted to hospital within 14 days after discharge.

**Table:** Baseline characteristics, diagnoses, and treatment of children hospitalized with EV-D68 respiratory illness in southwest Finland. August–September 2022*

Variable	Children with EV-D68, N = 56
Median age, years (IQR)	4.23 (2.42–6.15)
Sex	
F	19 (33.9)
M	37 (66.1)
Underlying condition, any	21 (37.5)
Asthma†	15 (26.8)
Neurologic condition	5 (8.9)
Premature birth, <37+0 gestational weeks	4 (7.1)
Other condition‡	3 (5.4)
Other virus§	8 (14.3)
Diagnosis	
Wheezing illness¶	44 (78.6)
Pneumonia	9 (16.1)
Upper respiratory tract infection	3 (5.4)
Treatment	
Intensive care unit admission	7 (12.5)
Respiratory support, any	32 (57.1)
Supplemental oxygen	22 (39.3)
High flow nasal oxygen	10 (17.9)
Invasive ventilation	0 (0)
Inhaled salbutamol	53 (94.6)
Systemic corticosteroids	42 (75.0)
Antibiotics	20 (35.7)
Mean length of stay in the hospital, days (SD)	2.75 (2.05)
Readmission#	1 (1.8)

*Values are no. (%) unless otherwise indicated. IQR, interquartile range; SD, standard deviation. †Confirmed or suspected asthma. ‡Cardiovascular condition, cystic fibrosis, or hypothyroidism. §Rhinovirus, n = 7; SARS-CoV-2, n = 3; and human bocavirus, n = 3. Including 3 patients who had 3 or 4 viruses. ¶Bronchiolitis, wheezing bronchitis, or exacerbation of asthma. #Within 14 days after discharge.

We detected another respiratory virus, in addition to EV-D68, in 8 (14.3%) children. Because other respiratory viruses can affect the clinical picture, we performed a sensitivity analysis of the clinical characteristics, excluding children with a co-detected virus (Appendix Table). We determined the clinical characteristics of 48 children with EV-D68 as the only detected virus to be similar to all study children.

## Conclusions

The EV-D68 outbreak we studied started rapidly and was exceptionally intense but was of short duration in our area. Other parts of Finland also reported an increased number of children hospitalized with wheezing illnesses during the same timeframe. As in earlier reports, we found that the clinical picture of EV-D68 infection in hospitalized children was most often a respiratory illness characterized by wheezing and respiratory distress. In that respect, EV-D68 resembles rhinoviruses more than it does other enteroviruses (*13*).

Compared with wheezing illnesses caused by other viruses, the median age of children with EV-D68–associated illness was somewhat high (4.2 years), and a large proportion of children had a severe illness treated at the intensive care unit. Some children were simultaneously positive for another respiratory virus, but clinical manifestations were similar among those with EV-D68 only and those with co-detected viruses. Co-detection of >2 respiratory viruses is common in children. Although cases in this outbreak included 1 child diagnosed with encephalitis and no cases of acute flaccid myelitis, EV-D68 can cause acute flaccid myelitis and other neurologic illnesses. A study of a larger population would be needed to determine the occurrence of rare manifestations.

Active surveillance for EV-D68 is important because of its epidemic occurrence, continuous antigenic evolution (*14*), and potential to cause severe respiratory illnesses and serious neurologic conditions. Rapid multiplex RT-PCR methods are increasingly used for microbiologic diagnostics of respiratory tract infections, but these methods do not provide virus type, and some methods do not discriminate between rhinoviruses and enteroviruses. Our findings support virologic laboratory testing, especially in severe clinical cases, to determine the enterovirus type.

Our study included only 1 large pediatric hospital in Finland, and the number of EV-D68 cases is probably underestimated because of the short study period; enterovirus testing of only children negative in the screening test for SARS-CoV-2, influenza A and B, and RSV; and use of either triplex or 16-plex RT-PCR as the primary test for enterovirus. If we had used both tests or the highly sensitive triplex test for all cases, sensitivity might have been higher. We were not able to determine the lineage of genotyped EV-D68 viruses based on partial sequencing of VP1 only.

In summary, we observed a rapid onset of an EV-D68 epidemic in southwest Finland in August 2022. Most children who needed hospital treatment had a respiratory illness characterized by acute wheezing and respiratory distress. Given the virus’ ability to cause acute flaccid myelitis and other neurologic illnesses, continuing surveillance for EV-D68 is needed.

Appendix Additional information for enterovirus D68 outbreak in children, Finland, August–September 2022.
