# Supplementary material for: Enterovirus D68 Outbreak in Children, Finland, August–September 2022
Source: Emerg Infect Dis. 2023 Jun;29(6):1258–61. doi: 10.3201/eid2906.221795 (PMC10202852; doi:10.3201/eid2906.221795)
Supplement: Appendix — Additional information for enterovirus D68 outbreak in children, Finland, August–September 2022. [file 22-1795-Techapp-s1.pdf]

Article DOI: http://doi.org/10.3201/eid2906.221795

EID cannot ensure accessibility for supplementary materials supplied by authors. Readers who have difficulty accessing supplementary content should contact the authors for assistance.

Enterovirus D68 Outbreak in Children, Finland, August–September 2022

Appendix

Virologic Methods

For virus identification, nucleic acids were extracted from the samples using NucliSense easyMag (BioMerieux) automated extractor. Prior to extraction swabs in dry test tubes were suspended in phosphate buffered saline. Routine testing for EV was performed using either a laboratory developed triplex RT-PCR assay detecting EV, rhinovirus, and RSV or Allplex Respiratory Panels 1–3 (Seegene) including EV as one of the targets. The laboratory developed triplex assay was set up using SensiFAST Probe One-Step master mix with earlier described primers and probes (*1*,*2*), and Mic PCR instrument (Bio Molecular Systems) was used for the amplification. Allplex assays were run on CFX96 cycler (Bio-Rad Laboratories) according to manufacturer's instructions.

We first subjected specimens positive for EV to typing based on partial EV VP1 gene sequencing after seminested PCR amplification (*3*). That revealed a high prevalence of EV-D68, but the amplification succeeded in specimens with high virus load (low Ct value) only. Therefore, we applied an RT-PCR assay with EV-D68 VP1 gene-specific primers (forward, GAACCAGAAGAAGCCATACAAACTC and reverse, ATCTCAGCATCAAATCTAAGGTATGTG) designed during an EV-D68 outbreak in 2014 (*4*). The assay was performed with SuperScript III Platinum One-Step qRT-PCR kit (ThermoFisher) supplemented with 1X EvaGreen Dye (Biotium) using Mic PCR instrument. Since a part of the specimens remained untyped, we subjected them to a nested amplification method for the VP4/2 gene region as follows. First, cDNA was generated and amplified for 10 cycles using SuperScript III Platinum One-Step qRT-PCR kit with outer primers (*1*,*5*). Then, the sequencing amplicon was generated on a Rotor-Gene 3000 cycler (Qiagen) in 35 cycles using Platinum SuperFi II

Master Mix (ThermoFisher) with 1X EvaGreen Dye and inner primers (*6*). Amplicons with typical amplification and melting curves were treated with ExoSAP (ThermoScientific). Sequencing in both directions was obtained from Eurofins Genomics. EV type was called according to high similarity with type sequences as compared with Basic Local Alignment Search Tool (https://blast.ncbi.nlm.nih.gov).

References

1. Österback R, Tevaluoto T, Ylinen T, Peltola V, Susi P, Hyypiä T, et al. Simultaneous detection and differentiation of human rhino- and enteroviruses in clinical specimens by real-time PCR with locked nucleic Acid probes. J Clin Microbiol. 2013;51:3960–7. PubMed https://doi.org/10.1128/JCM.01646-13

2. Toivonen L, Schuez-Havupalo L, Rulli M, Ilonen J, Pelkonen J, Melen K, et al. Blood MxA protein as a marker for respiratory virus infections in young children. J Clin Virol. 2015;62:8–13. PubMed https://doi.org/10.1016/j.jcv.2014.11.018

3. Nix WA, Oberste MS, Pallansch MA. Sensitive, seminested PCR amplification of VP1 sequences for direct identification of all enterovirus serotypes from original clinical specimens. J Clin Microbiol. 2006;44:2698–704. PubMed https://doi.org/10.1128/JCM.00542-06

4. Poelman R, Schuffenecker I, Van Leer-Buter C, Josset L, Niesters HG, Lina B; ESCV-ECDC EV-D68 study group. European surveillance for enterovirus D68 during the emerging North-American outbreak in 2014. J Clin Virol. 2015;71:1–9. PubMed https://doi.org/10.1016/j.jcv.2015.07.296

5. Savolainen C, Mulders MN, Hovi T. Phylogenetic analysis of rhinovirus isolates collected during successive epidemic seasons. Virus Res. 2002;85:41–6. 10.1016/s0168-1702(02)00016-3 PubMed https://doi.org/10.1016/S0168-1702(02)00016-3

6. Linsuwanon P, Payungporn S, Samransamruajkit R, Posuwan N, Makkoch J, Theanboonlers A, et al. High prevalence of human rhinovirus C infection in Thai children with acute lower respiratory tract disease. J Infect. 2009;59:115–21. PubMed https://doi.org/10.1016/j.jinf.2009.05.009

Appendix Table. Baseline characteristics, diagnoses, and treatment of children hospitalized with EV-D68 respiratory illness in southwest Finland in August–September 2022, excluding cases with simultaneous detection of another respiratory virus*

Variable	Children with EV-D68, N = 48
Median age, years (IQR)	4.23 (2.67-6.03)
Sex	
F	15 (31.3)
M	33 (68.8)
Underlying condition, any	19 (39.6)
Asthma†	14 (29.2)
Neurologic condition	4 (8.3)
Premature birth <37+0 gestational weeks	4 (8.3)
Other condition‡	3 (6.3)
Diagnosis	
Wheezing illness§	39 (81.3)
Pneumonia	6 (12.5)
Upper respiratory tract infection	3 (6.3)
Treatment	
Intensive care unit admission	4 (8.3)
Respiratory support, any	27 (56.3)
Supplemental oxygen	21 (43.8)
High flow nasal oxygen	6 (12.5)
Invasive ventilation	0 (0)
Inhaled salbutamol	45 (93.8)
Systemic corticosteroids	35 (72.9)
Antibiotics	15 (31.3)
Mean length of stay in the hospital, days (SD)	2.52 (1.79)
Readmission¶	1 (2.1)

*Values are no. (%) unless otherwise indicated. IQR, interquartile range; SD, standard deviation.
†Confirmed or suspected asthma.
‡Cardiovascular condition, cystic fibrosis, or hypothyroidism.
§Bronchiolitis, wheezing bronchitis, or exacerbation of asthma.
¶Within 14 days after discharge.
